# The National Village Health Guide Scheme in India: lessons four decades later for community health worker programs today and tomorrow

**DOI:** 10.1186/s12960-019-0413-1

**Published:** 2019-10-28

**Authors:** Rachel J. Strodel, Henry B. Perry

**Affiliations:** 1 Yale University, New Haven, CT United States of America; 2 Department of International Health, Health Systems Program, Bloomberg School of Public Health, Johns Hopkins University, Baltimore, MD United States of America

**Keywords:** Community health workers, Primary health care, Community health, India, Village Health Guides

## Abstract

**Background:**

Based in part on the success of India’s early community health worker (CHW) programs, the Government of India launched in 1977 a national CHW scheme—the Village Health Guides (VHGs)—to provide preventive, promotive, and basic curative care to rural populations. Although this program had promising origins in smaller demonstration projects, it failed to deliver the hoped-for impact at scale and was abandoned. Based on extensive evidence and experience, the World Health Organization and the World Health Assembly have strongly endorsed the value of national CHW programs and their integration into national health systems. Surprisingly, given the scale and importance of the VHG program and its pioneering nature as a national CHW program, little has been published describing this experience. This article is the second in a series that focuses on critical issues that face the effectiveness of large-scale CHW programs.

**Case presentation:**

Several systemic factors emerge as main contributors to the failure of the VHG Scheme, namely, a lack of support from the formal health sector, an overly hasty implementation of the scheme, and poor communication between the government and health centers about the role of the VHGs. The remuneration structure and the VHG selection process were at the root of the program’s shortcomings at the implementation level.

**Conclusion:**

National CHW schemes are an increasingly important tool for achieving universal health coverage and ending maternal and child deaths by 2030. Although the VHG Scheme was initiated over 40 years ago, the lessons described in this case highlight important considerations to help both current and future large-scale CHW programs avoid the same pitfalls.

## Key messages

- India’s attempt to take to national scale a community health worker program based on several small-scale projects—such as the Comprehensive Rural Health Project at Jamkhed, Maharashtra—was a bold and visionary step to address unmet health needs in the country.
- Unfortunately, little is known about the history of this program because it quietly disappeared. There is little documentation of the implementation of the program or evaluation of it after it was underway. This paper seeks to summarize the available information about this program.
- The history of the Village Health Guide Scheme, along with substantial national experience since in India and beyond, points to the importance of careful planning by engaging health system actors at multiple levels, engaging the community, integrating the program with the health system, and obtaining buy in for long-term political and financial support to ensure sustainability and long-term effectiveness.

## Background

A significant challenge on the road to Health for All, as called for in the Declaration of Alma-Ata in 1978 [1], is expanding small, successful community health worker (CHW) programs to achieve the same impact at scale. Program implementers have often assumed that if a smaller CHW program has been successful, then the same results can be replicated at a national level. This, however, is often not the case. Between the initial small-scale project and the broad, national scheme, effectiveness can be lost in translation, and the impact of the program can be diluted.

The Village Health Guide (VHG) program, initiated across India in 1977, offers an example of a national CHW program that failed to deliver the same impact as the grassroots projects from which it drew its inspiration. Although the Government of India launched the VHG Scheme over 40 years ago, it is valuable to examine the factors that led to the collapse of the Scheme, especially as CHW programs continue to emerge, adapt, and expand around the globe. This analysis uses a case study approach to trace the history of the VHG Scheme within the broader context of the Indian health system. We highlight the political barriers, implementation challenges, and other considerations that often threaten national CHW programs, with the aim of shedding light on how other CHW programs can avoid the same pitfalls in the future. This article is part of a series of articles that focus on issues of expanding and strengthening large-scale community health worker programs [2, 3].

## Methods

We searched PubMed, Scopus, and Google Scholar with the search terms “Village Health Guide”, “Community Health Guide”, “Health Guide”, and “Community Health Volunteer” for any documents pertaining to the VHG Scheme in India. We also contacted persons who are knowledgeable about the program and interviewed some of them. We also identified appropriate references from other articles about the VHG Scheme.

## Case presentation

### Historical context

The VHG Scheme emerged from a health system in which the wellbeing of rural peoples had been largely ignored. British colonial rule in India gave little attention to rural health services since the health system established by the British was developed to cater to the needs of military and bureaucratic officials [4]. At the time of independence, only approximately 10% of the Indian population had access to appropriate medical care [5].

In the years leading up to independence in 1947, this widespread neglect resulted in grassroots efforts calling for greater access to promotive, preventive, and curative services. Several committees articulated this vision for improving the health of the people of India through community-based initiatives. In 1940, the National Health (Sokhey) Sub-Committee of the National Planning Committee called for a CHW program that would provide one worker for every 1000 village population to provide basic curative and preventive services [6]. Like the Sokhey Subcommittee, the Bhore Committee advocated for the integration of preventive and curative services in its influential report published in 1946 [7]. This report made clear that community engagement was necessary to transform the existing health system: “The closer the health service can be brought into contact with the people whom it serves, the fuller will be the benefit it can confer on the community” [7]. Together, these declarations seemed to signal a shift toward addressing the longstanding neglect of the health of Indian people, particularly those living in rural areas.

Despite the ambitious visions of justice and community engagement articulated by the Bhore report and Sokhey Subcommittee, the health system continued to reflect India’s deep-seated inequalities. Physicians comprised an elite class of professionals incentivized to become highly specialized and practice in urban areas [5]. Although the Government of India adopted the Bhore Committee’s recommendation of establishing primary health care (PHC) centers, the actual implementation of these health centers languished. Rural health centers designed to serve 40 000 individuals were stretched to cover 100 000. Even though the catchment areas of these centers were 15–20 km, utilization was feasible only for those within 4–5 km of the center since the only available transport was by foot or cart [8].

Throughout the 1960s and 1970s, there was little progress toward improving health services in rural areas. During the 1960s, the government trained extension workers to conduct home visits and deliver specific, targeted interventions (e.g., family planning services or immunizations). However, with only one worker per 10 000–25 000 population, these paramedics were unable to reach all individuals in their catchment area [9]. Communities were also generally unengaged in the preventive activities the workers promoted [9]. As a result, high fertility and high infant mortality continued unabated, as did health disparities between urban and rural regions. In the early 1970s, the crude death rate in rural regions was nearly double that of urban regions [10].

To address these issues, the Ministry of Health and Family Welfare launched the Health and Medical Education Committee (commonly referred to as the Srivastava Committee) in 1974. The aim of the Srivastava Committee was to align medical training with the country’s needs and establish guidelines for a new group of health workers to improve coordination between multipurpose workers and medical officers [11]. This Committee’s report would later become the foundation for the planning of the VHG Scheme. The Committee’s vision was to “make the community self-sufficient in the provision of simple, promotive, preventive and curative health services” [12].

In its report, the Srivastava Committee recommended principles to govern the development of a new CHW program. The workers in the program would include existing health workers who provide outreach (e.g., auxiliary nurse midwives), but the program would also train community members such as schoolteachers and educated unemployed women with the skills necessary to become CHWs. In order to prevent these new CHWs from becoming an extension of the bureaucracy, the Committee recommended that they be neither remunerated nor supervised by the state, and instead be “free to work with the community on the basis of the trust and confidence they can generate” [11]. The primary aim of this new cadre would be to increase access at the community level to preventive, promotive, and curative care services and to create a link between rural communities and the formal health sector.

When the Janata Party (with its stronger orientation to Gandhi’s principles than Nehru’s [13]) defeated the Congress Party in the 1977 election, the new government began to draw up plans to institute the health worker program outlined by the Srivastava Committee. The government also drew inspiration from the CHW programs of several smaller projects, particularly the Jamkhed Comprehensive Rural Health Project (CRHP) and CRHP’s co-founder, Rajanikant Arole, who was a government advisor for planning the Rural Health Scheme (Raj Arole, personal communication, 1997) and 13 other projects in India highlighted at a symposium in 1976 [14].

In the first few years of the new government, leaders felt pressure to regain the confidence of India’s rural populations and saw the implementation of a CHW program as a step that would demonstrate a commitment to their wellbeing. Rapidly implementing this CHW program would also reduce the risk of the medical community organizing against the reform, as it had done previously [8]. However, this haste resulted in a program that deviated in many important ways from the Srivastava Committee’s original vision [8].

The first important difference between the Committee’s recommendations and the actual program was the decision to pay the VHGs a small monthly “honorarium” to cover expenses—Rs. 200 during training and Rs. 50 (approximately US$25 and US$6 in 1978 currency, respectively or US$100 and US$24 in 2019 currency) per month post-training, in addition to providing Rs. 50 worth of medicines and supplies per month [13]. Although the Srivastava Committee recommended that the government not remunerate the new cadre of VHGs, paying the new workers would help legitimize the new government: the Janata Party could point to the program as a success in providing jobs for the educated but unemployed youth in India’s rural population [8].

In addition, the selection criteria for VHGs deviated from the Srivastava Committee’s recommendations. Smaller pilot projects, including the Jamkhed CRHP [15], found success with training female CHWs, and the Srivastava Committee suggested training one male and one female worker per 5000 population [11]. However, the program planners did not specify any criteria regarding the gender of the VHGs beyond advising that the workers be female when possible. This guideline was hardly ever upheld, and 75–94% of VHGs were male [12, 16–18], making it in effect a part of the political patronage system [19]. The selection of predominantly male workers based more on political considerations than on a desire to serve ultimately became a major factor undermining the effectiveness of the VHG Scheme.

The program plan included several additional guidelines outlining the selection, training, and duties of the VHGs. Community members were responsible for selecting their own VHG [20]. If the community felt that the VHG was not performing satisfactorily, it could replace the worker but would have to raise the funds for training the new VHG [9]. After 3 months of training at the local PHC center, the VHG was expected to work 2–3 h per day [8, 9, 20]. The VHGs were tasked with identifying cases of communicable diseases such as malaria, tuberculosis, and leprosy; administering first aid; treating minor ailments; helping paramedics in their work related to immunization, family planning, and maternal and child health; rallying the community around sanitation and hygiene; and generally promoting health education [14, 20]. If a particular case required medical skills beyond the training of a VHG, the VHG was expected to refer it to the PHC center, the District Hospital, or another specialized facility [13]. The government intended for communities to be responsible for supervising the VHG, but the PHC center would provide technical support and hire an additional medical officer to support the new workers [9].

### Program implementation and evaluation (1977–2002)

On October 2, 1977, less than 7 months after the Janata Party had been elected, the VHG Scheme was officially instituted. The scheme was first called the “Community Health Worker (CHW) Scheme, but in 1979 was renamed the Community Health Volunteer (CHV) Scheme,” and finally in 1981 designated the “Village Health Guide Scheme.” [12, 13]. Here, we use the term VHG to refer to this group of CHWs throughout the life of the program.

The first wave of training involved 741 PHC centers in 28 districts throughout India and approximately 8000 new VHGs [13]. Eighteen months later, there would be approximately 66 000 functioning VHGs across India [19]. By 1980, 150 000 CHWs had been trained, reaching one third of India’s rural population [21]. This figure would rise to 400 000 by the end of 1987 [13, 18]. As originally intended by the Srivstava Committee, the aim was to train one VHG for every 1000 people.

Soon after implementation began, the challenges of implementing a grassroots program through a top-down approach directed by a central government bureaucracy became apparent. The government issued several statements announcing the new program, but communities, health personnel, and even the VHGs received little clarity as to how the responsibilities of the new VHGs would fit into the existing heath system. Although the communities were supposed to play a key role in selecting their own VHGs, in reality this task was often delegated to just a handful of individuals, including medical officers at the PHC centers and the village leadership (members of the Village Panchayats) [13, 20]. In some cases, political parties and elected representatives took advantage of the selection process to advance their own interests.

When physicians in Bengal spoke up about this exploitation of the system, the selection of VHGs became standardized and controlled by district-level officials to prevent this type of interference [8]. As a result, communities—which were intended to be central participants in VHG selection—were sidelined.

Once selected, the training that VHGs received at their local PHC centers failed to provide them with the skills necessary to carry out their assigned responsibilities. The PHC centers themselves were overcrowded, and groups of trainees sometimes had to sit outside in the PHC center courtyard when there was no room for their class to meet [8]. The legacy of aggressive family planning campaigns through these centers during the 1960s and 1970s left few resources to be dedicated toward strengthening other health services. The instructors training the VHGs were often unaware of the overall goals and methods of the VHG Scheme, and only one half of the VHG trainees received training manuals [21, 22]. Merely 3% of VHGs received their medical kits during training, further impeding the learning of new skills [8]. Though the curriculum included practical skills such as latrine building and water purification, it did not include education on broader social factors affecting health. In reality, it was as if the VHGs were being trained as assistants for the PHC center rather than as community advocates [12].

VHG Scheme evaluations in the late 1970s and early 1980s reflect these deficiencies and an emphasis on curative care. A survey conducted by the National Institute of Health and Family Welfare in 1979 found that VHGs responded correctly to only 20–30% of questions about patient referral, disease prevention, and emergency care [21]. This same evaluation found that VHGs responded correctly less than 30% of the time to questions assessing knowledge of disease prevention; conditions requiring referral to higher-level facilities; emergency treatments; and general preventive, promotive, and curative services [21]. Following this evaluation, little was done to address these shortcomings. The VHGs also failed to constitute a link between the community and the PHC centers, referring on average fewer than two patients to the PHC centers every 2 weeks [8]. A study in the Mysore district of Karnataka found that although 80% of VHGs correctly identified malaria symptoms, only 20% knew how to make oral rehydration solution to treat diarrhea [21]. Communities themselves perceived the VHGs primarily as a provider of curative care. In one study, 74% of the population thought that the most important task of the VHGs was to treat minor ailments, while only 0.4% said that community health education was most important [23].

All evaluations carried out revealed a high level of contact of VHGs with the population they were serving and a high level of satisfaction with the curative care that they provided. A 1979 national evaluation found that 57% of respondents had had contact with a VHG and a similar evaluation in 1984 found that 54% had had contact [22]. One study from Karnataka reported VHGs were seeing on average 8.6 patients per day [21]. A 1988 evaluation in Barasia block of Bhopal district [24] found that 82% of those interviewed had utilized a VHG and that 72% were fully satisfied with the VHG’s services. The main cause of dissatisfaction was lack of drugs. In a 1998 evaluation in the Punjab State, for instance, 70% of households interviewed had obtained care from a VHG and 65% had received a home visit from a VHG [12]. Other evaluations carried out during this period reported similar high levels of contact of VHGs with the population and of satisfaction with the work of the VHGs [25, 26].

The author of the Bhopal district evaluation concluded “it is evident that … the role of the V.H.G. [as] essentially that of change agent to educate the community on health, nutrition, immunization, maternal and child health and environmental sanitation has not been fulfilled so far” [24]. In other words, the VHGs were largely not fulfilling their mission to engage their communities in preventive activities.

To make matters worse, the VHG stipend became the crux of several issues plaguing the program. Providing a monthly stipend to each VHG created a substantial burden for the central government. In 1979, the National Development Council asked the state governments to cover half the cost of the scheme, causing several states to terminate the program [13]. The stipend also impacted how the VHGs viewed their responsibilities. Although the remuneration was small, it led communities, health system personnel, and VHGs themselves to regard VHGs as another level of government employees [20]. The payments shifted the role of the VHG away from that of a community advocate, educator, and link to the formal health system. Instead, VHGs’ duties became centered on basic curative care and tasks assigned to them by medical personnel.

### Evolution of the Village Health Guide Scheme

After the Janata Party government dissolved toward the end of 1979 and the Congress Party returned to power, the government mandated that the cost of the scheme be equally borne by the states, and most states dropped the program [13]. The VHG Scheme was abandoned for a year. In 1981, the Congress Party government resuscitated the program as a centrally funded scheme, mandating that villages form health committees to guide, monitor, and supervise the work of the CHWs (who were then named VHGs) [13]. By 1982, VHG Schemes were operating in all States of India except for three, which had their own alternative scheme [27]. Throughout the 1980s and 1990s, the government neglected the VHG Scheme and did little to address its well-established failures. In 1986, the government attempted to phase out male VHGs and re-orient the scheme toward maternal and child health [20]. However, this led male VHGs to unionize and petition against their removal since they considered themselves government employees [17]. The amount of the honorarium also became a source of tension between VHGs and the government. By 2001, at least 23 legal cases had been brought to trial to raise the honorarium—but as VHGs were technically volunteer workers, none succeeded in court [20].

From 1997 to 2001, a high-level committee from the Government of India evaluated the VHG Scheme to determine whether the program had achieved its aims and if the Government should continue supporting the scheme in the future [20, 28]. Based on the recommendations of this committee, the government formally terminated its financial support for the VHG Scheme in April 2002, although states were encouraged to continue funding the scheme out of their own budgets [20].

## Discussion

### Lessons learned

The VHG Scheme highlights several important lessons for CHW programs today. Although the challenges of scaling up CHW programs have been described elsewhere, the VHG Scheme provides a unique glimpse into how a country’s politics and health system norms can shape the scale-up of CHW programs. Because the program was initiated over 40 years ago, there is also opportunity to examine how India’s CHW schemes have evolved and incorporated lessons from failure into their contemporary programs.

The failure of the VHG Scheme was a systemic one, as shown in Table 1. The seeds of its downfall began with motives geared to gain quick political support for a newly elected government but without a sustained, long-term commitment. The problems began with the hasty planning of the scheme, which prioritized portraying the new government in a good light over ensuring that the structural elements of the program were sound. It is important to note that political pressures will inevitably differ in different countries and eras: not all CHW programs will uniformly experience the same political urgency or lack thereof. Nonetheless, the pressures surrounding the design of the VHG Scheme resulted in hasty decision-making in crucial elements of the program, namely in establishing a stipend that could not be sustained and in recruiting almost exclusively male workers without a strong service orientation.

**Table 1:** Factors contributing to the failure of the Village Health Guide Scheme

Factor	Description
Hasty planning and scale-up	The rapid expansion of the scheme left little room for adaptation or iterative learning. Thus, the problems identified in early evaluations of the scheme were not sufficiently addressed.
Poor communication	Communication between the central government and the local communities about the scheme was limited. As a result, there was much confusion among communities and PHC centers surrounding the VHGs and the role they were meant to fulfill.
Distorted selection process	The Scheme’s planners intended for communities to select and supervise their own VHGs. However, this task was often guided by only a select group of community leaders and later by district-level officials (after patterns of political patronage became apparent). Furthermore, despite recommendations that there be an equal number of male and female workers, almost all the VHGs selected were male.
Lack of support from the health system	PHC centers were poorly poised to train and supervise a CHW cadre
Suboptimal supervision	In the theoretical outline of the scheme, VHGs were meant to be supervised by the community. In practice, however, this task was often delegated to the local PHC centers, deemphasizing the community-centered goals of the VHG program.
Lack of adequate logistical support	VHGs were often without needed supplies and medicines
Issues related to remuneration	Although the compensation that VHGs received was relatively small, it substantially altered the perception of their work. VHGs were regarded as government workers rather than community advocates and educators. In addition, the honorarium became unsustainable for the central government to fund in later years of the program.

In addition, the planners did not pay enough attention to the fact that the health system in which the VHGs would operate was apathetic, or even antagonistic, toward the type of preventive care, health promotion, and social mobilization that the VHGs would advocate. As a result, state officials did the bare minimum to implement the program, at times even opposing the scheme [22]. Very few PHC center staff members received instruction in how to train the new VHGs, leading the PHC centers to consider the VHGs as just another level of government worker. All this, plus weak communication from the central and state levels of the Ministry of Health and Family Welfare to the district level and the local PHC centers reduced community participation in the selection and monitoring of VHGs. Ultimately, the VHG Scheme’s weaknesses highlight the importance of clear communication and buy-in—from the central government all the way through the local PHC center.

The VHGs were intended to be a cadre tasked with a broad array of duties related to preventive, promotive, and curative services, as well as advocacy and education [13]. Due to poor communication between the central government and rural communities, the VHGs’ promotive and preventive roles fell by the wayside, and their work became centered on curative tasks. Weak coordination between the central government and state actors jeopardized even those curative responsibilities, with almost half of all VHGs lacking their essential drug kits in 1979 [21]. What began as a program with the potential to empower communities ultimately disintegrated because of inadequate support and insufficient changes in the structure of the existing health system.

It is interesting to note that the history of the VHG Scheme corroborates the existing literature on factors that enable or detract from the success of a CHW program scale-up. A seminal 1989 review of national CHW programs in Botswana, Colombia, and Sri Lanka concluded that all three programs suffered from “unrealistic expectations, poor initial planning, problems of sustainability, and difficulties in maintain quality” [29]. A systematic review by Pallas et al. found that the most salient enablers of successful CHW scale-ups and sustainability include consistent management and supervision of CHWs; recruitment of individuals from the communities they serve; integration of the CHWs with the larger health system; and strong government support for the CHWs—financially and politically [30]. This same review identified major barriers to scaling up, including insufficient incentives; weak CHW supervision; a lack of community support for the CHW; and poor reception of CHWs into the existing health system [30].

In our analysis, we observe that most of these barriers to CHW scale-up were present in the VHG Scheme, while many of the enabling factors were absent. Finally, a recent systematic review of existing reviews of CHWs [31] also emphasized the importance of community embeddedness (whereby community members have a sense of ownership of the program and positive relationships with the CHW), supportive supervision, continuous education, and adequate logistical support and supplies. The review also highlighted the importance of effective integration of the CHW program into the health system to bolster program sustainability and credibility as well as to clarify CHW roles. All of these features were deficient in the case of the VHG Scheme.

### The Village Health Guide Scheme through the current lens of Universal Health Coverage, Ending Preventable Child and Maternal Deaths, and the continued quest of Health for All

The VHG Scheme was an important early attempt to extend basic and essential health care at scale in India, and the lessons learned have been valuable for India and are valuable today in the current global context of striving for Universal Health Coverage, Ending Preventable Child and Maternal Deaths, and the continuing the quest of Health for All. The VHG Scheme was eventually followed in 2005 by the Accredited Social Health Activist (ASHA) Program, introduced on a national scale in India by the government’s National Rural Health Mission, which drew on lessons from the VHG program [32]. Now, there are almost 1 million ASHA workers, all of whom are female [32]. There is a well-planned supervisory and management structure for the ASHA workers with efforts to embed them both in the community and in the broader health system (Smisha Agarwal personal communication, 6 June 2019). The program is much more effective and enjoys broad political national and local support as well as strong ongoing financial support for the program (Rajani Ved, personal communication, 28 June 2016).

Although India still has a long journey ahead in making its national CHW programs fully effective, other countries such as Brazil, Niger, Ethiopia, Rwanda, Nepal, and Bangladesh have been quite successful in implementing national CHW programs to enable the achievement of national health goals [32]. There is now a resurgence of interest in national CHW programs based on the successes of the CHW programs in these countries and on the demonstrated effectiveness of CHW programs and community-based primary health care [33–35]. As such, it is anticipated that CHW programs will be an important component of achieving the global health goals for 2030—achieving Universal Health Coverage [36] and ending Preventable Child and Maternal Deaths [37].

Many of the lessons learned from the VHGs have now been incorporated into recently adopted guidelines of the World Health Organization for integrating CHW programs into health systems that were released in 2018 [38, 39]. In May 2019, the World Health Assembly passed a historic, first-ever resolution on CHWs that recognizes their essential role in primary health care and their need to be well-integrated into and supported by health systems [40].

In an ideal world, national CHW programs would emerge through a series of discussions and negotiations at various levels of the health system and with civic society actors, including associations of health professionals. A governance system is needed that involves ongoing negotiations among health system actors and communities [41]. Positive CHW program outcomes require community embeddedness along with effective integration with health systems to enable “program sustainability and credibility, clarify CHW roles, and foster collaboration between CHWs and higher-level health system actors” [31].

The VHG program was one of the first efforts to scale up a CHW program. The lessons from this experience could not be more relevant than for today, when there is global recognition of the potential contributions of CHWs to strengthening health systems. It is clear that there is a need to avoid the mistakes of the VHG Scheme in order to enable CHW programs to reach their full potential.

## Conclusion

Scaled-up CHW programs can be powerful tools to address the needs of underserved populations, but they are not magic bullets, nor a “panacea for weak health programs” [31]. As the case of the VHG Scheme demonstrates, these programs require a government willing to make a long-term commitment to a CHW program that is closely integrated with the health system. When appropriate guidelines are set forth and when political leaders are invested in their success, CHW programs can make a lasting impact on health and wellbeing. Otherwise, however, these programs can become another broken facet of struggling health systems without bringing the community-level change they are designed to promote.

## Data Availability

Not applicable.

## Abbreviations

ASHA: Accredited Social Health Activist
CHW: Community health worker
PHC: Primary health care
VHG: Village Health Guide
